# Diagnostic test accuracy of assessment tools for detecting delirium in patients with acute stroke: commentary of a systematic review

**DOI:** 10.12968/bjnn.2022.18.Sup5.S18

**Published:** 2022-11-01

**Authors:** Malabika Ghosh, Oliver Hamer, James Hill

**Affiliations:** 1 Lancashire Teaching Hospitals, NHS Foundation Trust, UK; 2 University of Central Lancashire (UCLan), Preston, UK

**Keywords:** Delirium, Stroke care, Diagnostic assessment, Measurement instruments, Psychometrics Systematic review

## Abstract

Delirium is a common presentation after acute stroke. Post-stroke delirium is related to poor recovery, higher rates of mortality, falls, and longer hospital stays. Delirium can lead to challenging behaviour such as anger, aggression, and confusion. As such, it is important to identify delirium promptly for early management and to reduce the negative impact on post-stroke recovery and outcomes. An important aspect of identifying delirium depends on the use of efficient, easy to use and validated assessment tools. A wide range of tools are available, although it is not known how accurately they can identify post-stroke delirium. This article critically appraises a systematic review which identified delirium screening tools for patients with acute stroke, and summarised their accuracy.

## Introduction

Delirium is a common complication of acute stroke with an incidence affecting one in four patients (Shaw et al, 2019a). Post-stroke period prevalence (within 10 days of admission) of delirium is common with incidence of 10-28% (Shi et al, 2012). More recently, the period prevalence (within 6 days of admission) has been reported to be 17% to 28% (Shaw et al, 2019b). Risk factors of post-stroke delirium include older age, previous cognitive impairment, pre-stroke dementia, dehydration, severe stroke, and infection (Shaw et al, 2019b). Hospitalised, elderly patients are especially susceptible to risk factors that lead to delirium, including immobilisation and the use of antipsychotic medication (Shaw et al, 2019a).

Delirium can lead to challenging behaviour such as anger, aggression, and confusion (Kowalska et al, 2020). Post-stroke delirium is related to poor recovery, higher rates of mortality, falls and longer hospital stays (Bauernfreund et al, 2018; Hshieh et al, 2018), with patients being 4.7 times more likely to die in hospital (Shi et al, 2012). Early detection of delirium is crucial as it helps to identify acute triggers, giving early access to recommended treatment pathways and supports risk management such as prevention of falls and distress. As such, it is important to identify delirium promptly for early management to reduce the negative impact on post-stroke recovery and outcomes. Several studies have targeted delirium prevention in at-risk patients (Inouye et al, 2014; Skrobik 2009), however much of this work has been outside of stroke. That said, there has been an acknowledgement among stroke research that there is need for careful planning, using targeted staffing and resources. Stroke is an established, predisposing, and precipitating cause of delirium, however almost one third of cases are preventable (Shaw et al, 2019). Hence, it is advocated that all stroke patients are screened for delirium for effective prevention and early management (McManus et al, 2007). However, screening is not always conducted because of time restraints associated with stroke care (McManus et al, 2007).

An important aspect of identifying delirium includes the use of efficient, user friendly and validated assessment tools (Andorra et al, 2022). There is widespread acknowledgement in literature regarding the use of standardised assessment tools in delirium (Helfand et al, 2021). A wide range of tools are available to identify delirium (Helfand et al., 2021). These assessment tools have not yet been comparatively assessed for validity and reliability in an acute stroke population (Mansutti et al, 2019). Thus, a systematic review by Mansutti et al (2019), aimed to identify delirium screening tools for patients with acute stroke and to summarise their accuracy.

## Aim of commentary

This commentary aims to critically appraise the methods used within the systematic review by Mansutti et al (2019), and expand upon the test accuracy findings in the context of clinical practice.

## Methods

The systematic review conducted a restrictive search of three databases from inception to September 2018: Medline, the Cumulative Index to Nursing and Allied Health Literature (CINAHL) and Scopus. Further to the database searches, the reference lists of included reports were screened for additional studies. Studies were eligible for inclusion if they evaluated tools detecting delirium among patients with acute stroke, were diagnostic test accuracy studies, written in English and were published up to September 2018. Studies were excluded if they were not conducted in the acute phase of patient stroke (first 48 hours to the following two weeks), evaluated tools aimed at screening other cognitive issues in patients with acute stroke (e.g., dementia, cognitive decline), analysed associations between post-stroke delirium and other risk factors, and publications only detailing a protocol.

A thorough screening process was undertaken in which two authors independently screened titles, abstracts, and full text articles. Data extraction was undertaken by two authors using a study-specific pro forma which had been piloted (analysed two studies). Disagreements within the process of study selection were discussed and resolved by a third author. A comprehensive risk of bias assessment was independently conducted by two authors using the QUADAS-2 tool. The systematic review analysed test accuracy data for six tools that detected delirium among patients with acute stroke: The 4-Assessment Test for delirium (4AT), the Confusion Assessment Method for the Intensive Care Unit (CAM-ICU), the Abbreviated Mental Test (AMT-10), the Abbreviated Mental Test- short version (AMT-4), the Clock Drawing Test (CDT) and the Glasgow Coma Scale (GCS). The tools were compared against two gold standard measurement instruments for the purpose of establishing criterion validity: the Diagnostic and Statistical Manual of Mental Disorders criteria (DSM) and the Confusion Assessment Method (CAM). Delirium was largely detected by neurologists, neuropsychiatrists, or physicians.

## Results

Following duplicate removal, 98 papers were identified for screening of which four studies were included in the review. These four studies assessed the accuracy of seven different/versions of delirium diagnostic assessment tools. These included 4AT, CAM-ICU, AMT-10, AMT-4, CDT, GCS and COG4. All four studies used a similar inclusion criterion, used a consecutive sampling method, and had a small sample size ranging from 73 to 129 patients. The majority of studies were judged to have major concerns regarding both patient selection and the referencing standard. This meant that the population of interest may not be appropriately represented within the studies, and that the comparator test used to verify the accuracy of the tool may have not been validated previously.

Of the three studies which assessed the 4AT tool, the sensitivity (true positive percentage) ranged from 100% to 90% and specificity (truly negative percentage) ranged from 65% to 86%. One study reported that the 4AT tool had an internal consistency of 0.80 using Cronbach alpha (Good internal consistency).

One study reported that the CAM-ICU had a sensitivity of 76% (95% confidence interval (CI): 55% to 91%), specificity of 98% (95% CI: 93 to 100) Inter-rater reliability (the extent to which two or more rates agreement) 0.94 Kappa (95% CI: 83 to 100) (Almost Perfect agreement) and likelihood ratio of 0.47 (95% CI: 27 to 83). The remaining five tools were assessed by a single study of which AMT-10 and the shortened version AMT-4 had a similar sensitivity (76% & 75%) and specificity (61% & 61%). CDT and COG4 also had similar levels of sensitivity (67% & 70%) and specificity (38% & 44%). GCS had a sensitivity of 17% and specificity of 81%.

## Commentary

The Joanna Briggs checklist for systematic reviews and research synthesis was used to critically appraise the quality of the research article (Aromataris et al, 2015). The article satisfied 9 out of the 11 criteria on the checklist. The systematic review only searched Medline, CINAHL and Scopus databases, so it may not have exhaustively covered evidence available outside these databases. It is possible that studies may have been missed which has the potential to impact on the estimates given in this review. It was also unclear from the review whether the authors had considered the issue of publication bias and taken specific steps to mitigate this. Despite these inconsistencies, it was deemed that this systematic review provided an accurate and comprehensive synthesis of the available studies that addressed the question of interest.

This systematic review compared the accuracy of several delirium assessment tools used in post-stroke delirium (Mansutti et al 2019). When selecting a post-stroke delirium tool, the findings from this review suggests the 4AT tool may be the most appropriate option for identifying patients with delirium given its high sensitivity. In a clinical setting, it is likely the 4AT tool will correctly identify more than 9 out of 10 stroke patients suffering delirium. However, it is also important to note that when using this tool, somewhere between 1 to 4 people may be misdiagnosed of having delirium which may result in additional workload. There is some rationale to support this recommendation that the 4AT tool may be the most appropriate tool to identify delirium, given that 4AT tool is already a standard tool in clinical practice and has been endorsed in national guideline documents (e.g., Scottish Intercollegiate Board) (Health improvement Scotland, 2019). Furthermore, the 4AT tool has advantages compared to other tools due to its brevity (takes less than 2 minutes to complete) and ability to detect low arousal as one of the early signs of delirium (Davis et al, 2019). The use of the 4AT tool has also been recommended for standardising illness severity for acutely ill patients (presenting to hospital) by the Royal College of Physicians (2020).

If 4AT tool is not available, the CAM-ICU may provide slightly lower levels of sensitivity in that 8 out of 10 people with delirium being correctly identified and only 1 person may be incorrectly diagnosed of not having delirium. However, there is less certainty of these estimates of sensitivity and precision due to the limited evidence. Additionally, when selecting these a tool, it is also important to be aware that some tools have been specifically designed and validated for different populations. For example, the CAM-ICU is a shorter version of the CAM (Confusion Assessment Method used for older adults) which was validated for use with critically ill patients within intensive care units. However, this focus on the specific population does have its advantages of rapid administration (Ely et al 2001) and no requirement for verbal communication from the patient (Thomason et al 2005). It also has benefits in that it uses a detailed protocol which can be followed by any member of a multidisciplinary team, without extensive training (Miranda et al, 2018).

Healthcare professionals may also want to consider other factors such as availability of patient history and arousal levels when selecting a tool for use in clinical practice (European Delirium Association 2014; Inouye et al 2014). In scenarios whereby collateral history is unavailable for the patient or when arousal levels are low (as is the case in many cases of delirium), the 4AT tool may be the most appropriate choice of tool because of its validation with a range of patients (Jeong et al. 2020). Given the limitations of these instruments within clinical practise, health professionals need to carefully consider the population, setting, level of staff training and condition of the patient when selecting a measurement tool to assess delirium in stroke patients.

As is clear from research evidence, none of the delirium assessment tools have been designed specifically for stroke. Most of the tools available also have a wide variation in sensitivity and specificity which could suggest the confounding impact of moderating factors (e.g., the need for additional training in the use of tools or the setting in which a tool is used). Given, the high incidence of post-stroke delirium, more research is needed to develop tools dedicated to diagnosing post-stroke delirium and measuring its impact, such as on cognitive impairment, behavioural change, and functional decline.

## CPD reflective questions

- Do multidisciplinary teams working in stroke have the requisite knowledge and skills to detect and measure delirium using validated tools?
- Does post-stroke delirium identification and management benefit from a well-designed stroke specific, validated tool?
- Do multidisciplinary teams give due importance to the management of post-stroke delirium and its effect on behaviour, function, and other outcomes?

## Key Points

- Out of the tools assessed, the 4-Assessment Test (4AT) was identified to have high levels of sensitivity and precision, followed by the Confusion Assessment Method for the Intensive Care Unit (CAM-ICU) for measuring post-stroke delirium.
- Health professionals need to carefully consider the population, setting, level of staff training and condition of the patient when selecting a measurement tool to assess delirium in stroke patients.
- Further research is needed to develop measurement tools to assess stroke specific delirium and its effect on challenging behaviour, cognitive and functional outcomes.
